# Phylogeny of Algal Sequences Encoding Carbohydrate Sulfotransferases, Formylglycine-Dependent Sulfatases, and Putative Sulfatase Modifying Factors

**DOI:** 10.3389/fpls.2015.01057

**Published:** 2015-11-26

**Authors:** Chai-Ling Ho

**Affiliations:** Department of Cell and Molecular Biology, Faculty of Biotechnology and Biomolecular Sciences, Universiti Putra MalaysiaSerdang, Malaysia

**Keywords:** algae, carbohydrate sulfotransferases, sulfatases, phylogeny, sulfatase modifying factors

## Abstract

Many algae are rich sources of sulfated polysaccharides with biological activities. The physicochemical/rheological properties and biological activities of sulfated polysaccharides are affected by the pattern and number of sulfate moieties. Sulfation of carbohydrates is catalyzed by carbohydrate sulfotransferases (CHSTs) while modification of sulfate moieties on sulfated polysaccharides was presumably catalyzed by sulfatases including formylglycine-dependent sulfatases (FGly-SULFs). Post-translationally modification of Cys to FGly in FGly-SULFs by sulfatase modifiying factors (SUMFs) is necessary for the activity of this enzyme. The aims of this study are to mine for sequences encoding algal CHSTs, FGly-SULFs and putative SUMFs from the fully sequenced algal genomes and to infer their phylogenetic relationships to their well characterized counterparts from other organisms. Algal sequences encoding CHSTs, FGly-SULFs, SUMFs, and SUMF-like proteins were successfully identified from green and brown algae. However, red algal FGly-SULFs and SUMFs were not identified. In addition, a group of SUMF-like sequences with different gene structure and possibly different functions were identified for green, brown and red algae. The phylogeny of these putative genes contributes to the corpus of knowledge of an unexplored area. The analyses of these putative genes contribute toward future production of existing and new sulfated carbohydrate polymers through enzymatic synthesis and metabolic engineering.

## Introduction

Sulfates are found in algal proteins, carbohydrate, sulfolipids, and low molecular weight sulfated compounds (DeBoer, 1981). Many algae were reported to be rich sources of sulfated polysaccharides with biological activities (Hernandez-Sebastia et al., 2008). Sulfated fucans from brown algae and sulfated galactans from green and red algae have been reported to be potent anticoagulant agents (Pomin and Mourão, 2008). Some of these algal sulfated polysaccharides such as agar, agarose, and carrageenan, constitute the major component of algal extracellular matrix or cell wall, and have wide applications in food, cosmetics and pharmaceutical industries (McHugh, 2003).

Sulfur (normally in sulfate form) constitutes one of the nine essential macronutrients required by plants including algae (Yildiz et al., 1994). Sulfur assimilation in plants and algae begins with the activation of sulfate by ATP sulfurylase, which catalyzes the adenylation of sulfate to 5′-adenylylsulfate (APS). APS can either be phosphorylated by APS kinase or reduced by glutathione-dependent APS reductase. Both enzymes and pathways are important for cellular synthesis of sulfated and reduced sulfur compounds in algae, respectively (Gao et al., 2000). Sulfation is catalyzed by sulfotransferases (STs) which transfer a sulfuryl group (SO_3_) from 3′-phosphoadenosine 5′-phosphosulfate (PAPS) to a hydroxyl group of a substrate (Hernandez-Sebastia et al., 2008). In addition, activities of sulfatases which were assumed to be involved in the modification of sulfate moieties on sulfated polysaccharides have also been reported in various algae. The pattern and number of these substitutions not only affect the physicochemical/rheological properties of sulfated polysaccharides but also their biological activities (Opoku et al., 2006; Tuvikene et al., 2008).

Carbohydrate sulfotransferases (CHSTs) are of particular interest in algae because several genera of marine macroalgae synthesize sulfated polysaccharides that constitute the major component of their cell walls which chelate metallic ions and provide hydration to the cells. Mammals CHSTs are among the best characterized CHSTs. Most of them are Golgi-localized and membrane-bound, and are involved in the biosynthesis of sulfated oligosaccharides and glycosaminoglycans (Fukuda et al., 2001). In addition, CHSTs such as NodH and NoeE that are involved in the biosynthesis of nodulation factors have also been characterized from symbiotic rhizobacteria, *Sinorhizobium melioti* and *Rhizobium* sp. NGR234, respectively (Ehrhardt et al., 1995; Hanin et al., 1997). Characterization of algal candidate genes for CHSTs has not been reported.

Formylglycine-dependent sulfatase (FGly-SULF) (EC 3.1.5.6) belongs to a large protein family that catalyze the hydrolytic desulfation of sulfate ester and sulfamates from different sulfated substrates. These sulfated substrates include hydrophobic glucosinolates, steroids, tyrosine sulfates, amphiphilic sulfated carbohydrates found in glycosaminoglycans (GAGs), proteoglycans, glycolipids, and water-soluble mono- and disaccharide sulfates (Hanson et al., 2004). FGly-SULFs consist of a class of enzymes that share highly similar amino acid sequence (20–60% over the entire protein length), three-dimensional structure and catalytic site (Boltes et al., 2001; Hopwood and Ballabio, 2001).

The conserved catalytic site of FGly-SULFs consists a divalent metal ion located within a pocket in which substrates are bound, a highly conserved motif at the N-terminus (or “sulfatase signature”) which spans over a 12-mer linear sequence with a core motif C/S-X-P-X-R, and a unique active aldehyde residue, α-formylglycine (FGly) (Hanson et al., 2004). FGly is formed post-translationally by the oxidation of a cysteine (Cys) residue that is conserved in all eukaryotic and most prokaryotic sulfatases (Schmidt et al., 1995; Dierks et al., 1998b). Some bacterial species possess serine (Ser) residue instead of cysteine (Cys) residue at the same position of the catalytic site leading to the “Cys-type” or “Ser-type” prokaryotic sulfatases. The structural similarity amongst FGly-SULFs suggested that they shared a common ancestral gene (Meroni et al., 1996; Parenti et al., 1997).

Post-translational modification of Cys to FGly occurs at the endoplasmic reticulum at a stage the polypeptide is not yet folded into its native structure (Schirmer and Kolter, 1998). The enzyme that is involved in the post-translational modification of Cys to FGly in FGly-SULFs is known as sulfatase modifiying factor (SUMF) while the enzyme AtsB is responsible for the post-translational modification of bacterial Ser-type sulfatases (Dierks et al., 1998b; Schirmer and Kolter, 1998). SUMF1 was found to be responsible for the multiple sulfatase deficiency in human. SUMFs belong to a gene family that is highly conserved during evolution from bacteria to human (Dierks et al., 1997, 1998a; Landgrebe et al., 2003).

Despite the importance of these sulfated polysaccharides, the roles of CHSTs in their formation, and sulfatases in their modifications; little is known about the sequences and structures of algal CHSTs, FGly-SULFs and their SUMFs. In recent years, a few algal genomes have been fully sequenced (Armbrust et al., 2004; Merchant et al., 2007; Bowler et al., 2008; Cock et al., 2010; Bhattacharya et al., 2013; Collén et al., 2013) and can be used for the survey for algal candidate genes encoding algal FGly-SULFs and SUMFs. The aims of this study are to mine for sequences from the fully sequenced algal genomes and to infer their phylogenetic relationships to known CHSTs, FGly-SULFs, and SUMFs from other organisms.

## Materials and methods

### Mining of algal sequences encoding CHSTs, FGly-SULFs, and SUMFs

Search analyses for sequences encoding algal CHSTs, FGly-SULFs, and SUMFs across nine completed algal genomes, i.e., *Chondrus crispus, Porphyridium cruetum* (http://cyanophora.rutgers.edu/porphyridium/), *Cyanidioschyzon* merolae (http://merolae.biol.s.u-tokyo.ac.jp/blast/blast.html), *Ectocarpus siliculosus* (http://bioinformatics.psb.ugent.be/orcae/overview/Ectsi), *Thalassiosira pseudonana, Phaeodactylum tricornutum, Ostreococcus tauri, Chlamydomonas reinhardtii*, and *Volvox carteri* (JGI: http://genome.jgi-psf.org/); and algal ESTs/cDNAs from *Porphyra umbilicalis, P. purpurea* (http://dbdata.rutgers.edu/nori/blast.php), *P. yezoensis, Laurentia dendroidea, Galderia sulphuraria, Gracilaria changii* and *G. salicornia*; were performed using the BLASTX, BLASTP, or TBLASTX algorithms (Altschul et al., 1990). The search was performed using the known sequences encoding CHSTs, FGly-SULFs, and SUMFs from human and/or other eukaryotes, i.e., mouse, rat, yeasts (*Neurospora crassa, Kluyveromyces lactis, Schizosaccharomyces pombe, Debaryomyces hansenii, Yarrowia lipolytica*), *Drosophila melanogaster* and worm *Caenorhabditis elegans* (Sardiello et al., 2005). Homologous sequences from plants were also retrieved from Phytozome ver.3 (http://phytozome.jgi.doe.gov/). BLASTX and BLASTP analyses were performed on the retrieved sequences against the SwissProt database. Sequences that do not match with any sequences encoding CHSTs, FGly-SULFs, and SUMFs were removed upon the reciprocal search. Amino acid sequences that were incomplete without the translation start methionine and the sulfatase signature for FGly-SULFs were also discarded.

### Phylogenetic analyses

Multiple sequence alignment of CHSTs, FGly-SULFs, and SUMF amino acid sequences were performed with Clustal W (Chenna et al., 2003), respectively. Phylogenetic analyses were conducted in MEGA4 (Tamura et al., 2007) using the Neighbor-Joining method (Saitou and Nei, 1987) with a bootstrap test performed on 1000 random combinations of the sequence alignment (Felsenstein, 1985).

### Generation of sulfatase signature logos

Logo analyses for FGly-SULF sequences were performed at the Berkeley Structural Genomics Center (http://weblogo.Berkeley.edu/) to visualize the information content associated with each position of a given motif shared by related sequences. In the graphical representation, the conservation at each position (expressed in bits) is represented by the overall height of each position whereas the relative frequencies of the symbols within a position are indicated by the relative sizes of the symbols. The reported values were computed as the rate between the information content of the given position and the information content of varying positions within the motif.

## Results and discussion

In total, 83, 41, and 14 algal sequences encoding CHSTs, FGly-SULFs, and SUMFs were retrieved, respectively (Tables 1–**3**). Human CHSTs, FGly-SULFs, and SUMFs were used for the mining and also phylogenetic analyses in this study mainly because sequences from human were the best characterized in terms of sequence and functions compared to those from other organisms.

**Table 1:** **The algal sequences encoding CHSTs**.

**Species**	**UniprotKB**	**Accession numbers**	
		**Enesmbl genomes**	**Identifier**
**SULFOTRANSFER_1 SUPERFAMILY^a^**			
*Chlamydomonas reinhardtii*	–	Cre16.g660390; CHLREDRAFT_105941	CHLRE_660390
	A8JIC1	Cre04.g230732; CHLREDRAFT_180605	CHLRE_A8JIC1
	A8IZD0	Cre08.g376650; CHLREDRAFT_173868	CHLRE_A8IZD0
	A8IZI4	Cre09.g387250; CHLREDRAFT_157945	CHLRE_A8IZI4
	–	Cre09g393321	CHLRE_393321
	A8J7Y1	Cre09.g401960; CHLREDRAFT_151050	CHLRE_A8J7Y1
	–	Cre10.g455231	CHLRE_455231
	A8J6P1	Cre17.g725950; CHLREDRAFT_176016	CHLRE_A8J6P1
*Micromonas pusilla* CCMP1545	C1N5Y2	MicpuC2.EuGene.0000140184|53089;MICPUCDRAFT_53089	MICPU_C1N5Y2
	C1N7H0	MicpuC2.EuGene.0000150366|53691; MICPUCDRAFT_53691	MICPU_C1N7H0
*Micromonas* sp. RCC299	C1EI91	MicromonasRCC299fgenesh2_pg.C_Chr_15000139|104367; MICPUN_104367	MICSR_C1EI91
	C1E122	MicromonasRCC299est_cluster_kg.Chr_03_33_3199036:1|107876; MICPUN_107876	MICSR_C1E122
	C1EIX6	MicromonasRCC299est_cluster_kg.Chr_16_22_3203032:1|109625; MICPUN_109625	MICSR_C1EIX6
	C1FIP1	MicromonasRCC299EuGene.1200010448|63276	MICSR_C1FIP1
*Volvox carteri*	–	Vocar20000292m	VOLCA_20000292
	–	Vocar20005856m	VOLCA_20005856
	D8TZS8	Vocar220009015m; VOLCADRAFT_92489	VOLCA_D8TZS8
	–	Vocar20010382m	VOLCA_20010382
	D8UM37	Vocar20010341m VOLCADRAFT_101239	VOLCA_D8UM37
	D8U792	Vocar220010585m; VOLCADRAFT_118754	VOLCA_D8U792
	D8U789	Vocar20010758m; VOLCADRAFT_64836	VOLCA_D8U789
	D8U1T1	Vocar20012944m; VOLCADRAFT_93309	VOLCA_D8U1T1
	D8UC65	Vocar220015136m; VOLCADRAFT_107145	VOLCA_D8UC65
	D8THD3	Vocar20001230m; VOLCADRAFT _85926	VOLCA_D8THD3
*Ostreococcus tauri* (strain OTH95)	Q019P5	OSTTA_4|17079; OT_ostta05g01260	OSTTA_ Q019P5
*Ostreococcus lucimarinus* CCE9901	A4S6Z1	OSTLU_eugene.1400010008; OSTLU_27293	OSTLU_ A4S6Z1
	A4RZY0	OSTLU_eugene.0700010148; OSTLU_32551	OSTLU_ A4RZY0
*Thalassiosira pseudonana*	B8C3E4	Thaps3|5614|fgenesh1_pg.C_chr_5000656; THAPSDRAFT_5614	THAPS_B8C3E4
	B8BX55	Thaps3|268435|estExt_thaps1_ua_kg.C_chr_30076; THAPSDRAFT_268435	THAPS_B8BX55
	B5YNL3	Thaps3|7193|fgenesh1_pg.C_chr_7000576; THAPS_7193	THAPS_B5YNL3
	B8CAP5	Thaps3|24552|estExt_fgenesh1_pg.C_chr_120299; THAPSDRAFT_24552	THAPS_B8CAP5
	B5YMN2	Thaps3|6848|fgenesh1_pg.C_chr_7000231; THAPS_6848	THAPS_B5YMN2
	B8C826	Thaps3|7980|fgenesh1_pg.C_chr_9000148; THAPSDRAFT_7980	THAPS_B8C826
	B8C3T6	Thaps3|5757|fgenesh1_pg.C_chr_5000799; THAPSDRAFT_5757	THAPS_B8C3T6
	B8BTN5	Thaps3|261251|thaps1_ua_kg.chr_2000075; THAPSDRAFT_261251	THAPS_B8BTN5
	B8CF62	THAPSDRAFT_11656	THAPS_B8CF62
*Phaeodactylum tricornutum*	B7FVB9	Phatr2|45024|estExt_fgenesh1_pg.C_chr_50413; PHATRDRAFT_45024	PHATR_B7FVB9
	B7FU87	Phatr2|44473|estExt_fgenesh1_pg.C_chr_40276; PHATRDRAFT_44473	PHATR_B7FU87
	B7FQD0	Phatr2|43022|estExt_fgenesh1_pg.C_chr_10750; PHATRDRAFT_43022	PHATR_B7FQD0
	B7FXZ9	Phatr2|35253|fgenesh1_pg.C_chr_7000174; PHATRDRAFT_35253	PHATR_B7FXZ9
*Ectocarpus siliculosus*	–	Esi_0203_0066	ECTSI_20366
	D8LIC4	Esi_0210_0041	ECTSI_D8LIC4
	D7FST9	Esi_0239_0035	ECTSI_D7FST9
	D7FV63	Esi_0289_0025	ECTSI_D7FV63
	D7FWT3	Esi_0312_0029	ECTSI_D7FWT3
	D7G0M1	Esi_0411_0021	ECTSI_D7G0M1
	D7G187	Esi_0442_0008	ECTSI_D7G187
	D7G3W5	Esi_0535_0006	ECTSI_D7G3W5
	D7G3W4	Esi_0535_0003	ECTSI_D7G3W4
	D7G676	Esi_0729_0004	ECTSI_D7G676
	D8LJX4	Esi_0028_0006	ECTSI_D8LJX4
	D7G1W1	Esi_0046_0070	ECTSI_D7G1W1
*Chondrus crispus*	R7QLM0	CHC_T00008796001	CHOCR_ R7QLM0
	R7Q533	CHC_T00008762001	CHOCR_ R7Q533
**SULFOTRANSFER_2 SUPERFAMILY^b^**			
*Cyanidioschyzon merolae*	–	CMT454C	CYAME_CMT454C
	–	CMT456C	CYAME_CMT456C
*Chondrus crispus*	R7QLI6	CHC_T00008402001	CHOCR_R7QLI6
	R7QVP9	CHC_T00008846001	CHOCR_R7QVP9
	R7QL39	CHC_T00009100001	CHOCR_R7QL39
	S0F3I6	CHC_T00009000001	CHOCR_S0F3I6
	R7QUP3	CHC_T00008342001	CHOCR_R7QUP3
	R7QIL9	CHC_T00008834001	CHOCR_R7QIL9
	R7Q8D2	CHC_T00009431001	CHOCR_R7Q8D2^*^
*Porphyridium cruetum*	–	evm.model.contig_2146.5	PORCR_2146.5
	–	evm.model.contig_2275.7	PORCR_2275.7
	–	evm.model.contig_2279.13	PORCR_2279.13
	–	evm.model.contig_2493.4	PORCR_2493.4
	–	evm.model.contig_3392.4	PORCR_3392.4
	–	evm.model.contig_435.12	PORCR_435.12
	–	evm.model.contig_4476.16	PORCR_4476.16
	–	evm.model.contig_4476.7	PORCR_4476.7
	–	evm.model.contig_493.17	PORCR_493.17
	–	evm.model.contig_522.5	PORCR_522.5
	–	evm.model.contig_528.3	PORCR_528.3
	–	evm.model.contig_528.4	PORCR_528.4
	–	evm.model.contig_604.4	PORCR_604.4
*Ectocarpus siliculosus*	D7G2B9	Esi_0047_0111	ECTSI_D7G2B9
	D8LIV5	Esi_0023_0057	ECTSI_D8LIV5
*Thalassiosira pseudonana*	B8C7Y2	THAPSDRAFT_7935	THAPS_B8C7Y2
	B5YNS1	THAPS_7251	THAPS_B5YNS1
*Phaeodactylum tricornutum*	B7G559	Phatr2|47859|estExt_fgenesh1_pg.C_chr_150105; PHATRDRAFT_47859	PHATR_B7G559
	B7G557	Phatr2|47857|estExt_fgenesh1_pg.C_chr_150103; PHATRDRAFT_47857	PHATR_B7G557
	B7FTQ4	Phatr2|44325|estExt_fgenesh1_pg.C_chr_40090; PHATRDRAFT_44325	PHATR_B7FTQ4

a Algal CHSTs with pfam 00685;

b algal CHSTs with pfam 03567 except for one algal CHST with pfam 06990^*^.

### Algal CHST sequences

**Figure 1:**
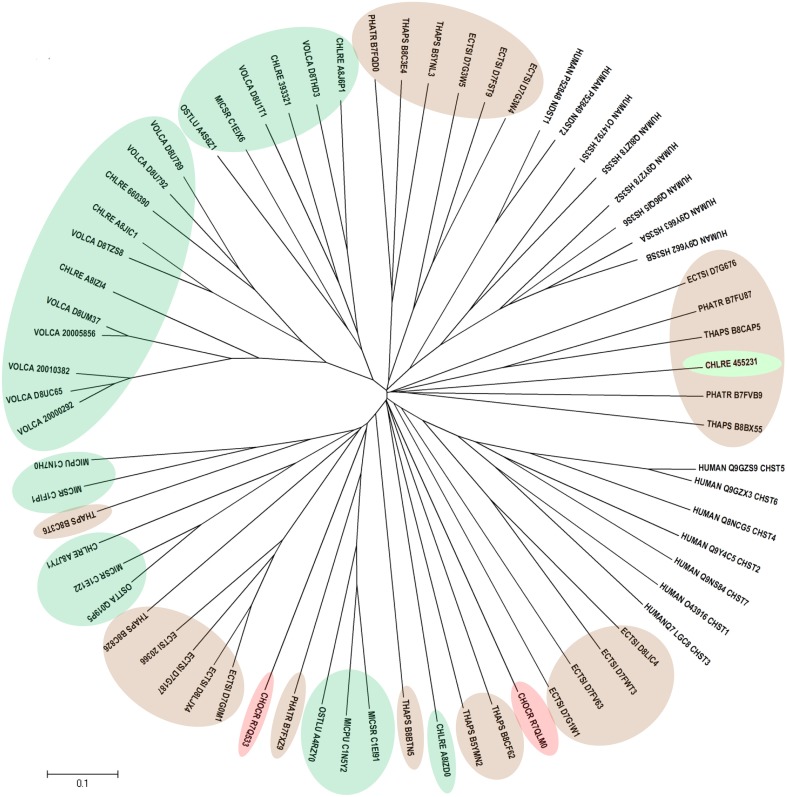
**Phylogenetic relationship of algal CHSTs in Superfamily Sulfotransfer_1 (with pfam domain 00685)**. The evolutionary history was inferred using the Neighbor-Joining method. The tree is drawn to scale, with branch lengths in the same units as those of the evolutionary distances used to infer the phylogenetic tree. Sequences from green, brown, and red algae are shown by respective colors. The identifier of the sequence starts with the species abbreviation followed by the UNIPROT/Genbank accession number and annotation wherever possible (Table 1). HUMAN, *Homo sapiens*; CHSTs 1-7, for Gal/N-acetylglucosamine/N-acetylglucosamine 6-O-STs; NDST, glucosamine N-deacetylase/N-ST or heparin sulfate STs; and HS3S1, 2, 5, 6, A and B, (heparan sulfate)-glucosamine 3-O STs.

**Figure 2:**
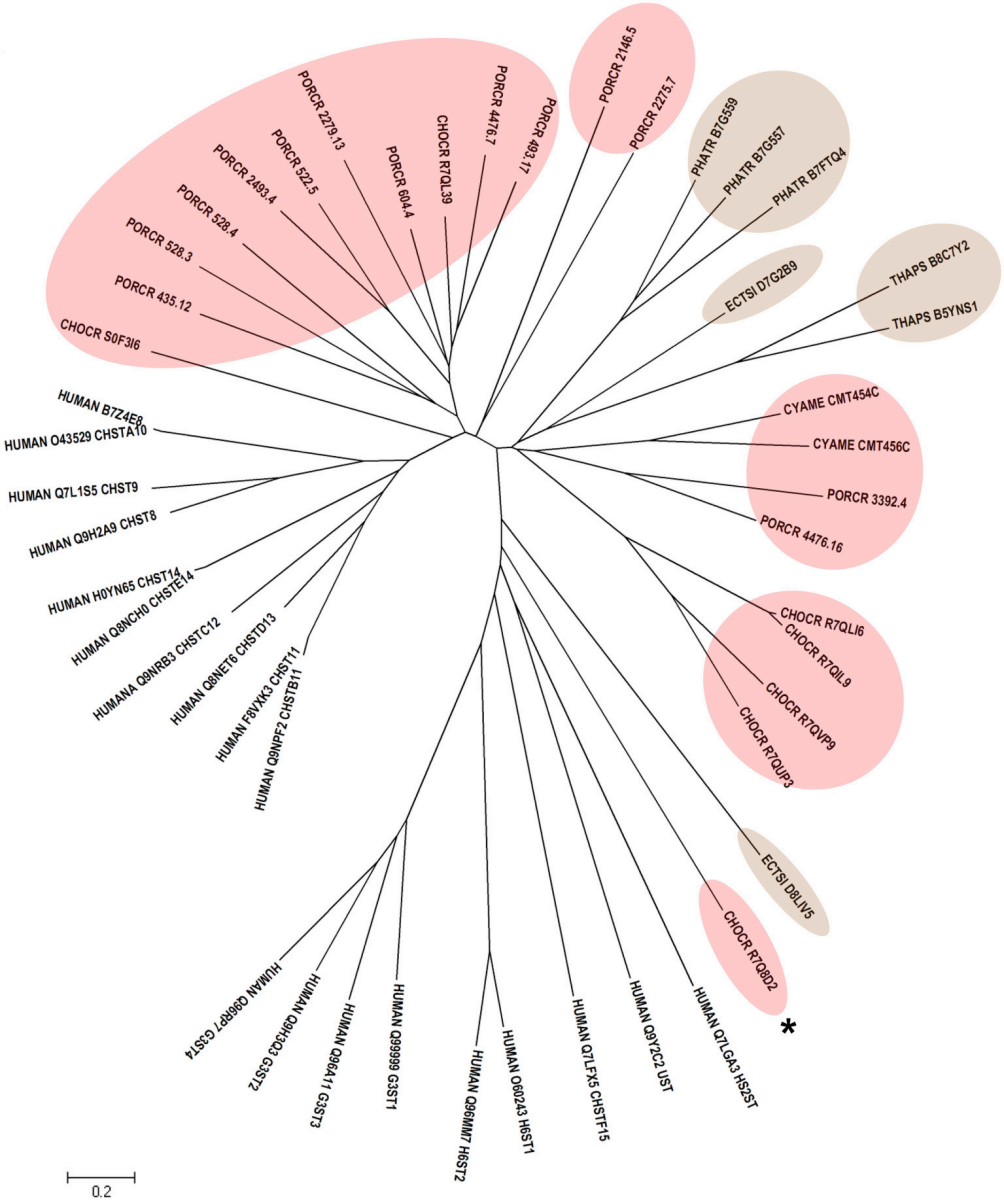
**Phylogenetic relationship of algal CHSTs in Superfamily Sulfotransfer_2 (with pfam domains 03567 and 06990)**. The evolutionary history was inferred using the Neighbor-Joining method. The tree is drawn to scale, with branch lengths in the same units as those of the evolutionary distances used to infer the phylogenetic tree. Sequences from brown and red algae are shown by respective colors. The identifier of the sequence starts with the species abbreviation followed by the UNIPROT/Genbank accession number and annotation wherever possible (Table 1). HUMAN, *Homo sapiens*; CHSTs 8-15, for N-acetylgalactosamine 4-O STs, and N-acetylgalactosamine 4-sulfate 6-O STs; H6ST 1-2, heparan sulfate 6-O-STs; HS2ST, heparan sulfate 2-O ST; UST, uronyl-2-O ST; G3ST1-4, galactose-3-O STs. ^*^ represents algal CHST with pfam 06990.

Human CHSTs can be divided into two groups based on the presence of two conserved domains for Superfamily Sulfotransferase 1 and 2, respectively (Figures 1, 2). All human CHSTs classified in the Superfamily Sulfotranferase 1 (CHSTs 1-7, mainly for Gal/N-acetylglucosamine/N-acetylglucosamine 6-O-STs; glucosamine N-deacetylase/N-ST or heparin sulfate STs, NDSTs; (heparan sulfate)- glucosamine 3-O STs, HS3S1, 2, 5, 6, A and B) were found to contain pfam 00685 for Sulfotransfer_1 domain, while most of those in the Superfamily Sulfotransferase 2 have pfam 03567 for Sulfotransfer_2 domain (CHSTs 8-15, for N-acetylgalactosamine 4-O STs and N-acetylgalactosamine 4-sulfate 6-O STs; heparan sulfate 6-O-STs, HS6ST 1-2; heparan sulfate 2-O ST, HS2ST and uronyl-2-O ST, UST) except for a few CHSTs such as galactose-3-O STs (G3ST1-4) that have pfam 06990 for Gal-3-O-Sulfotr domain. The findings on human CHSTs concur with the information published in the Interpro abstract for IPR005331 (www.ebi.ac.uk/interpro/) that Sulfotransfer_2 domain (pfam 03567) is present in a number of CHSTs that transfer sulfate to positions 3 (CHSTs 10), 4 (CHSTs 8, 9, 11 and 13; dermatan-4 ST, D4ST) and 6 (HS2ST, HS6ST, chondroitin-6 ST) of carbohydrate groups in glycoproteins and glycolipids. According to the Interpro abstract for IPR000863, Sulfotransfer_1 domain is found in flavonyl-3-STs, aryl STs, alcohol STs, and phenol STs. However, we found that many human CHSTs also contain this domain. The algal CHSTS (Table 1) were found to have either one of the pfams mentioned above or with no putative domain. All the green algal CHSTs were found to have pfam 00685 only while either pfam 00685 or pfam 03567 was found in the brown and red algal CHSTs. Only one red algal CHST from *C. crispus* (CHOCR_R7Q8D2) was found to have pfam 06990 (Figure 2, Table 1). The algal CHST sequences are generally very diverse. The use of phylogeney in assigning functions based on substrate specificity or pattern of sulfation requires further verification. Most of these algal CHSTs were clustered according to green, brown or red algae or even genera, except for a few clusters with green, red, and brown algal CHSTS (Figure 1). For examples, CHLRE 455231 was clustered among a group of brown algal CHSTs; THAPS B8C3T6 was in a group of green algal CHSTs; CHLRE A8IZD0 and CHOCR R7QLM0 were clustered with brown algal CHSTs; and CHOCR R7Q533 and PHATR B7FXZ9 were clustered with green algal CHSTs. These CHSTs could share similar functions in algae of different genera/species.

### Algal FGLy-SULF sequences

Sequences encoding putative algal FGly-SULFs were identified from complete green and brown algal genomes (Table 2). Although, sulfatase activities have been reported in a few red algae (Rees, 1961a,b; Wong and Craigie, 1978; Genicot-Joncour et al., 2009; Shukla et al., 2011; Qin et al., 2013; Wang et al., 2014), algal FGly-SULF was not retrieved from the red algal genomes i.e., *C. crispus* as reported by Collén et al. (2013), and red microalgal genomes from *Por. purpureum* (Bhattacharya et al., 2013) and *Cy. merolae* (Matsuzaki et al., 2004). Neither were these sequences detected among the available ESTs of red seaweeds from *P. yezoensis* (Nikaido et al., 2000; Kakinuma et al., 2006), *Griffithsia okiensis* (Lee et al., 2007) and *G. changii* (Teo et al., 2007). However, a few ESTs or incomplete cDNAs that are highly similar to sequences encoding FGly-SULFs were identified from *P. purpurea*. These sequences consist of partial coding sequences and share highly similar sequences to bacterial FGly-SULFs thus were not included in this analysis.

**Table 2:** **The algal sequences encoding FGly-SULFs**.

**Species**	**Accession numbers**		
	**UniprotKB**	**Enesmbl Genomes**	**Identifier**
*Thalassiosira pseudonana* (strain CCMP1335)	–	Thaps3|11324_fgenesh1_pg_C_chr_19c_29000010	THAPS_11324
	–	Thaps3|260259_thaps1_ua_pm_chr_19c_29000005	THAPS_260259
	B8LDP8	Thaps3|38351_e_gw1_19c_4_1; THAPSDRAFT_38351	THAPS_B8LDP8
	–	Thaps3|21474_estExt_fgenesh1_pg_C_chr_20778; (THAPSDRAFT_2824)	THAPS_21474
	B8BVG0	Thaps3|2824_fgenesh1_pg_C_chr_2000779; THAPSDRAFT_2824	THAPS_B8BVG0
	B5YNB4	Thaps3|7088_fgenesh1_pg_C_chr_7000471; THAPS_23517	THAPS_B5YNB4
*Phaeodactylum tricornutum* (strain CCAP 1055/1)	B7FQ28	Phatr2|32051_fgenesh1_pgC_chr_1000652; PHATRDRAFT_42934; Phatr3_J42934	PHATR_B7FQ28
	B7G541	Phatr2|24789_estExt_Genewise1C_chr_10620; PHATRDRAFT_47845	PHATR_ B7G541
	B7FQ28	Phatr2|52839_phatr1_ua_pmchr_1000076; PHATRDRAFT_42934	PHATR_ B7FQ28
	B7G541	Phatr2|38161_fgenesh1_pgC_chr_15000087; PHATRDRAFT_47845	PHATR_B7G541
*Ectocarpus siliculosus*	D7FLR5	Esi_0160_0032	ECTSI_D7FLR5
	D7G1A6	Esi_0444_0009	ECTSI_D7G1A6
	D7FLS5	Esi_0160_0052	ECTSI_D7FLS5
	D7G7Y4	Esi_0086_0031	ECTSI_D7G7Y4
	D7FKH5	Esi_0144_0037	ECTSI_D7FKH5
	D8LRL9	Esi_0069_0045	ECTSI_D8LRL9
	D7FUW5	Esi_0280_0019	ECTSI_D7FUW5
	D7FLR4	Esi_0160_0027	ECTSI_D7FLR4
	D7FLS1	Esi_0160_0043	ECTSI_D7FLS1
*Chlamydomonas reinhardtii*	A8ISJ6	ARS1, CHLREDRAFT_205496	CHLRE_A8ISJ6_ARS1
	Q9ATG5	ARS2, CHLREDRAFT_55757	CHLRE_Q9ATG5_ARS2
	A8IBH3	ARS3, CHLREDRAFT_140923	CHLRE_A8IBH3_ARS3
	P14217	ARS	CHLRE_P14217_ARS
	A8IB37	CHLREDRAFT_186203	CHLRE_A8IB37
	A8I963	CHLREDRAFT_111806	CHLRE_A8I963
	A8IB85	CHLREDRAFT_205499	CHLRE_A8IB85
	A8I8K3	CHLREDRAFT_166346	CHLRE_A8I8K3
	A8JFK7	CHLREDRAFT_153903	CHLRE_A8JFK7
	A8J863	CHLREDRAFT_192731	CHLRE_A8J863
	A8IT92	CHLREDRAFT_145838	CHLRE_A8IT92
	A8IT77	CHLREDRAFT_145830	CHLRE_A8IT77
	A8IT91	CHLREDRAFT_189474	CHLRE_A8IT91
	A8HPB7	CHLREDRAFT_189674	CHLRE_A8HPB7
*Volvox carteri*	D8TXL4	VOLCADRAFT_104983; ars1; EFJ47661	VOLCA_D8TXL4
	D8TUN6	VOLCADRAFT_120839; EFJ48739	VOLCA_D8TUN6
	–	Vocar20000600m; (VOLCADRAFT_120839)	VOLCA_20000600
	D8TUN4	VOLCADRAFT_90537; Vocar20000622m; EFJ48854	VOLCA_D8TUN4
	D8TSH9	VOLCADRAFT_59221; EFJ49375	VOLCA_D8TSH9
	–	Vocar20008567m; (VOLCADRAFT_ 86751)	VOLCA_20008567
	D8UFA8	Vocar20010817m; VOLCADRAFT_119669	VOLCA_D8UFA8
	D8TJI2	VOLCADRAFT_86751; EFJ52546-	VOLCA_D8TJI2

It is likely that the genes encoding FGly-SULF are absent from the genomes of red algae or at least in the red algal species examined. Since sulfate is not a limiting factor for marine algae that grow in seawater which has a high sulfate concentration (25–28 mM) compared to freshwater or land (10–50 μM) (Friedlander, 2001; Bochenek et al., 2013), it is possible that recycling of sulfate through FGly-SULFs may not be required. Furthermore, the biosynthesis of sulfated polysaccharides was proposed to be a possible result of physiological adaptation of macroalgae, marine angiosperms, and seagrasses (but not terrestrial plants) to marine environments (Aquino et al., 2005).

It is also possible that the red algal sulfatases belong to sulfatases other than the FGly-SULF type. Currently, three groups of sulfatases have been described: Group 1 which consists of the FGly-SULFs (Boltes et al., 2001); Group 2 with the Fe(II) α-ketoglutarate-dependent sulfatases (Müller et al., 2004); and Group 3 which consists of the zinc-dependent metallo β-lactamase superfamily or alkylsulfatases (Hagelueken et al., 2006). In addition, sulfatases (arylsulfatases) together with alkaline phosphatases and phosphoglycerate mutases were shown to belong to a superfamily of phospho-/sulfo-coordinating metalloenzymes that share the catalytic core of nucleotide pyrophosphatases/phosphodiesterases by homology searches and alignment-assisted mutagenesis (Gijsbers et al., 2001). The sulfatase genes may have also diverged to an extent that they cannot be readily identified using bioinformatic search tools. The red algal sulfatase could have novel sequences as reported for 12 sequences encoding putative D-galactose-2,6-sulfurylases I and II as revealed by the genome analyses of *C. crispus* (Collén et al., 2013). The galactose-2,6-sulfurylases I from *C. crispus* which share some identities to L-amino acid oxidase from *C. reinhardtii* (U78797) have no similarities to any reported sulfatases. Evidence on the enzyme activity of their recombinant proteins is crucial to show that they are indeed novel red algal sulfatases.

Sulfatase-like activities have also been reported previously in higher plants (Baum and Dodgson, 1957; Poux, 1966) although sequences encoding these enzymes have not been reported. Searching the complete plant genomes at the Phytozome revealed only one incomplete FGly-SULF-like sequence from *Ricinus cucumis* which contains a CSATR motif which resembles the sulfatase signature. However, this sequence was incomplete, short, and without introns. Further analyses revealed that similar sequences (orthologs) were absent in other plant species, thus was believed to be contaminated sequence from associated bacterial species.

**Figure 3:**
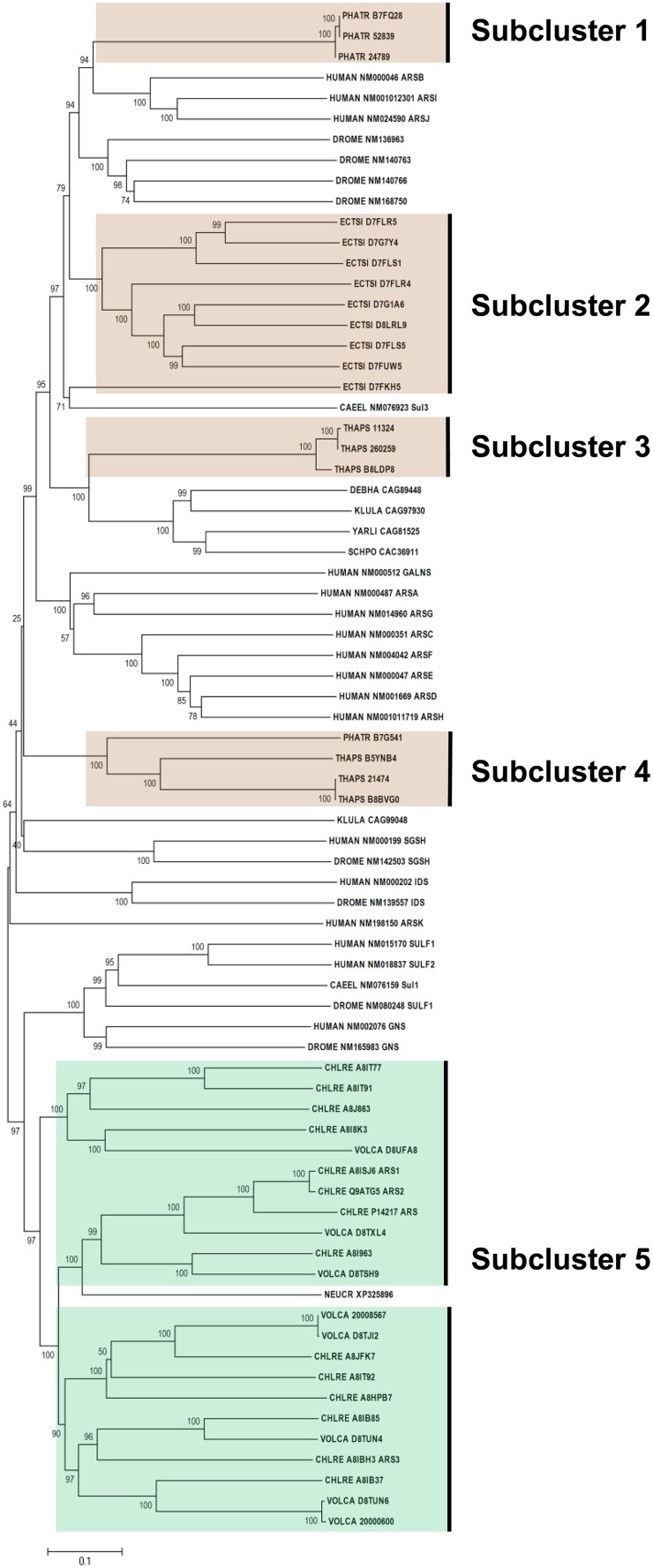
**Phylogenetic relationship of algal FGly-SULFs**. The evolutionary history was inferred using the Neighbor-Joining method. The percentage of replicate trees in which the associated taxa clustered together in the bootstrap test (1000 replicates) are shown next to the branches. The tree is drawn to scale, with branch lengths in the same units as those of the evolutionary distances used to infer the phylogenetic tree. Sequences from green and brown algae are shown by respective colors. The identifier of the sequence starts with the species abbreviation followed by the UNIPROT/Genbank accession number and annotation wherever possible (Table 2). HUMAN, *Homo sapiens*; CAEEL, *Caenorhabditis elegans*; DROME, *Drosophila melanogaster*; DEBHA, *Debaryomyces hansenii*; KLULA, *Kluyveromyces lactis*; YARLI, *Yarrowia lipolytica*; SCHPO, *Schizosaccharomyces pombe*; NEUCR, *Neurospora crassa;* ARS, arylsulfatase; GALNS, N-galactosamine-6-sulfatase; IDS, iduronate 2-sulfatase; and SGSH, N-sulfoglucosamine sulfohydrolase; Sulf, sulfatase; Sul, sulfatase.

Figure 3 shows the phylogeny of FGly-SULFs from human, yeasts, worm, fruitfly, and algae which has two main branches. The well characterized human FGly-SULFs were divided into two main branches with sulfatases SULF 1 and SULF 2, and glucosamine N-acetyl-6-sulfatase (GNS) in one branch while the remaining human FGly-SULFs (arylsulfatases, ARS A, B, C, D, E, F, G, H, I, J, K; N-galactosamine-6-sulfatase, GALNS; iduronate 2-sulfatase, IDS; and N-sulfoglucosamine sulfohydrolase, SGSH) are distributed in the other branch. The clustering of human FGly-SULFs may reflect their functions or substrate preference in general. The only two FGly-SULFs from worm were distributed one in each branch with SUL 1 in the same cluster as the human SULF 1 and SULF 2. The *D. melanogaster* SULF1, GNS, IDS, SGSH were grouped with their orthologs from human while another four uncharacterized FGly-SULFs formed a separate cluster which is unique for *D. melanogaster*. The FGly-SULF sequences from yeasts were clustered in the same branch except for that of ascomycetes *Neurospora crassa* which was found to be in a separate branch. It is likely that the FGly-SULFs from Saccharomycetes and Schizosaccharomycetes may have evolved after the divergence from Ascomycetes.

All the green algal FGly-SULFs (*Ch. reinhardtii* and *V. carteri*) were distributed in the same branch as human SULF 1, SULT2, and GNS, while all the brown algal FGly-SULFs were divided into subclusters in the other branch (Figure 3), implying that FGly-SULFs from these two groups of algae could have evolved from different origins or from the same origin which has diversified before speciation of brown and green algae. The green algal subcluster (Subcluster 5) consists of sequences from both *Ch. reinhardtii* and *V. carteri* thus implying that these sequences may have originated from the same ancestral FGly-SULF which could have existed before speciation. The brown algal sequences were divided into four subclusters: Subcluster 1 which consists of three sequences from *Ph. tricornutum* whereby each contains an extra C-terminus; Subcluster 2 with nine sequences from *E. siliculosus;* Subcluster 3 which consists of three sequences from *T. pseudonana* with a gap in pfam 00884 and closely related to yeast FGly-SULFs (except for the sequence from *N. crassa*); Subcluster 4 which consists of three sequences from *T. pseudonana*, and a sequence from *Ph. tricornutum*; and Subcluster 5 which contains mainly green algal FGly-SULFs. The existence of highly identical sequences from each species suggests duplication of FGly-SULFs upon speciation (Figure 3).

All algal sequences analyzed contain the sulfatase domain (pfam 00884), with a few of them bearing a gap within this domain, mainly those from diatoms (three from *T. pseudonana* in Subcluster 2 and one from *Ph. tricornutum* in Subcluster 4). However, the presence of gap within these sequences has little consequence in affecting their phylogeny compared to their similarity within the same species. In addition, sequences from *Ph. tricornutum* in Subcluster 1 contain an extra C-terminus.

**Figure 4:**
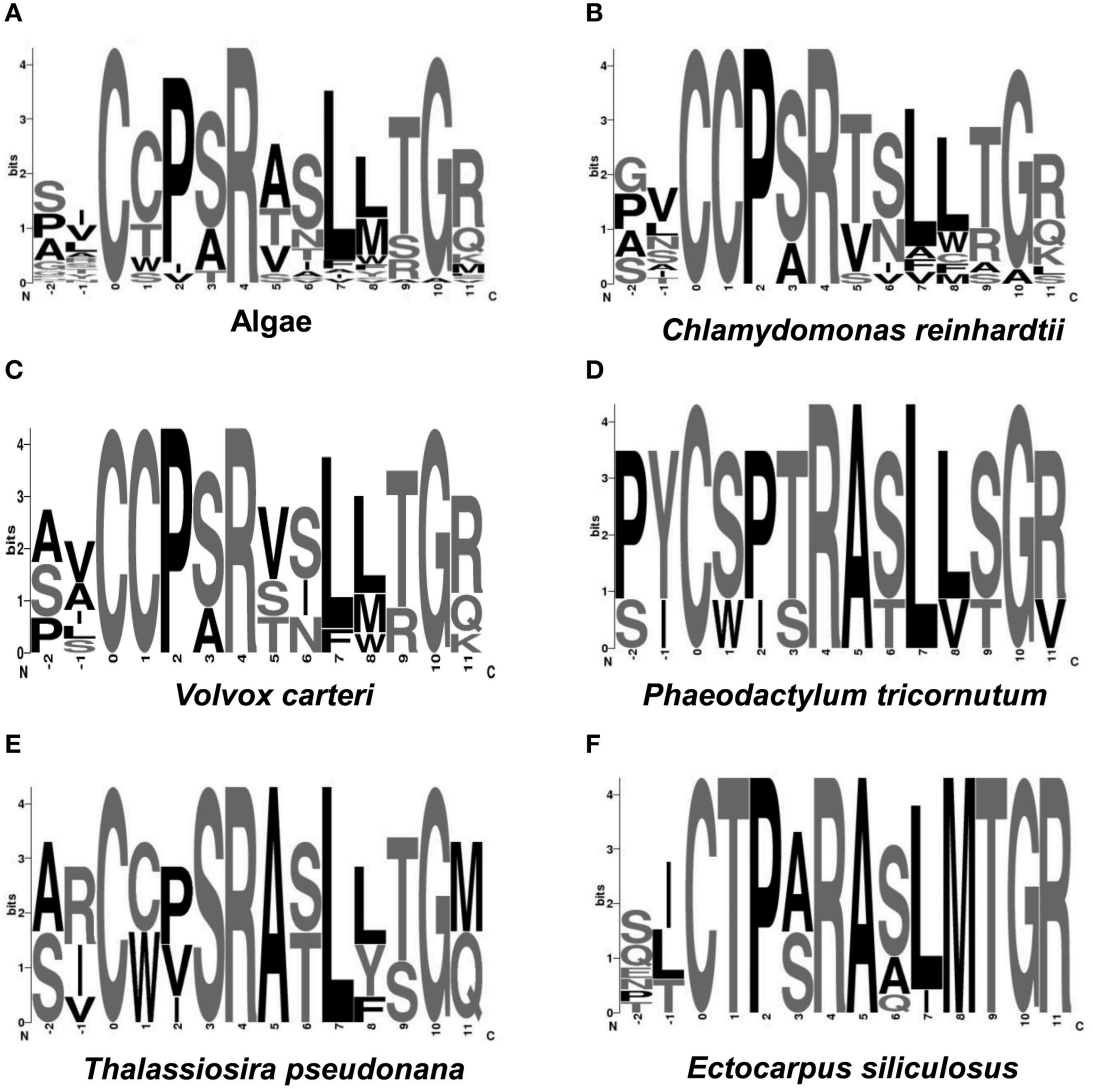
**Logo representation of the catalytic cores of algal FGly-SULFs**. The overall height of each column is proportional to the information content at that position, and within columns the conservation of each residue is visualized as the relative height of symbols representing amino acids. Position 1 indicates the residues directly involved in the enzymatic reaction. Position 1 of sulfatase cores indicates the amino acid (cysteine) to be modified into FGly. **(A)** Algae; **(B)** *Chlamydomonas reinhardtii*; **(C)** *Volvox carteri*; **(D)** *Phaeodactylum tricornutum*; **(E)** *Thalassiosira pseudonana*; **(F)** *E. siliculosus*.

The comparison of amino acid at the active sites of algal sulfatases showed that the green and brown algae share only two conserved residues (C-X-X-X-R) at the same positions (Figure 4A), which is less conserved compared to the core motif for human FGly-SULFs (C-X-P-S-R). Both *Ch. reinhardtii* and *V. carteri* share the same core motif: C-C-P-(S/A)-R (Figures 4B,C), while the brown algae have more diverse core motif (C-X-X-X-R; Figures 4D–F) whereby only the first C residue and the last R residue are conserved. Within the FGly-SULFs from each brown algal species, the core motif C-T-P-(A/S)-R is conserved among those from *E. siliculosus* (Figure 4F), while C-(S/W)-(P/I)-(T/S)-R and C-(C/W)-(P/V/I)-S-R were shared by those in *Ph. tricornutum* and *T. pseudonana*, respectively (Figures 4D,E). The A residue immediately after the core motif is highly conserved in brown algae (Figures 4D–F).

### Algal SUMF and SUMF-like sequences

**Figure 5:**
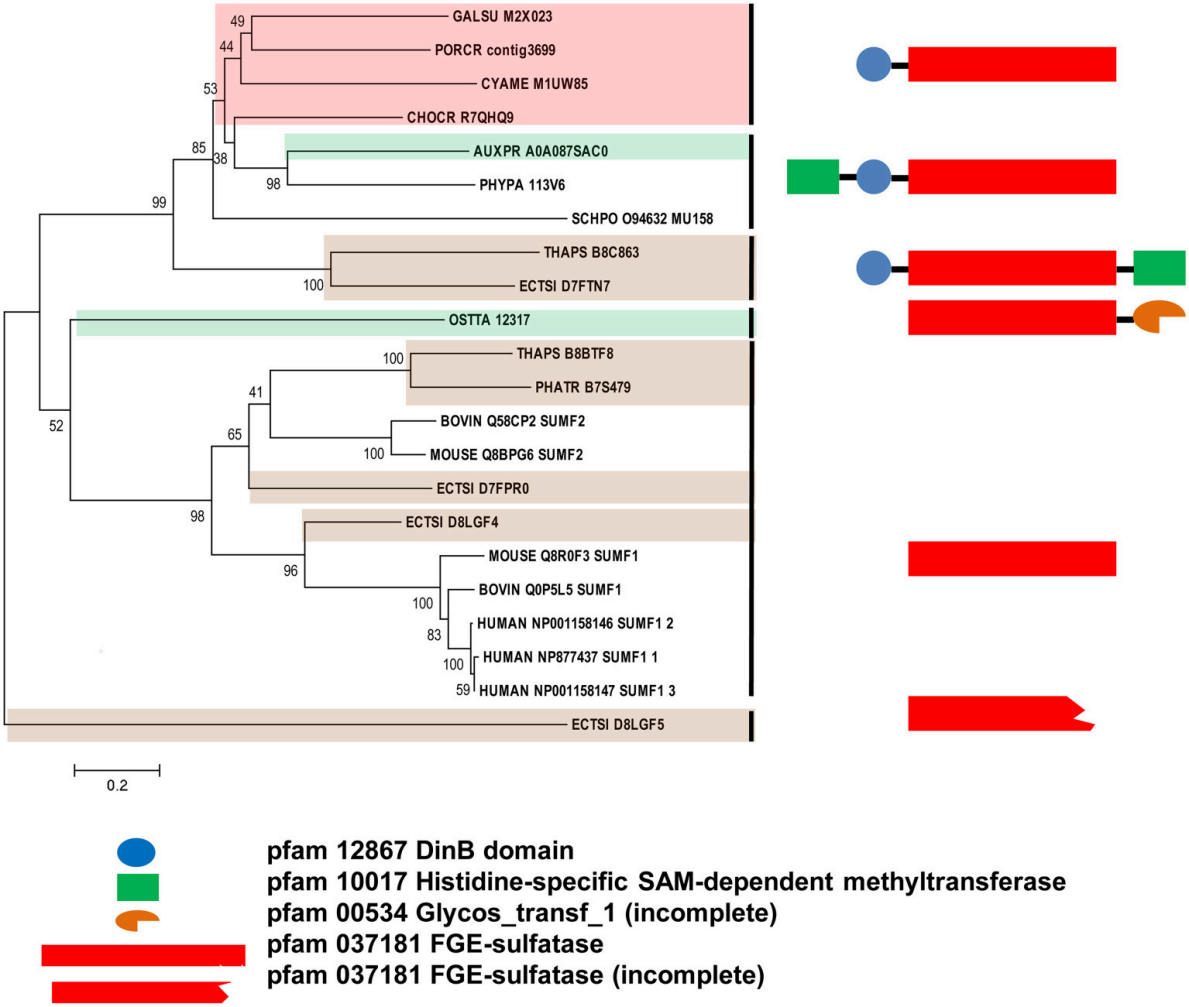
**Phylogenetic relationship of algal SUMFs and SUMF-like sequences**. The evolutionary history was inferred using the Neighbor-Joining method. The percentage of replicate trees in which the associated taxa clustered together in the bootstrap test (1000 replicates) are shown next to the branches. The tree is drawn to scale, with branch lengths in the same units as those of the evolutionary distances used to infer the phylogenetic tree. The identifier of the sequence starts with the species abbreviation followed by the UNIPROT/Genbank accession number and annotation wherever possible (Table 3). HUMAN, *Homo sapiens*; BOVINE, *Bos taurus*; MOUSE, *Mus musculus*; PHYPA, *Physcomitrella patens;* SCHPO, *Schizosaccharomyces pombe*. Sequences from green, brown, and red algae are shown by respective colors. The structure and pfam domains of each group are shown on the right panel.

Since the sulfatase signature was identified in all algal FGly-SULF sequences (Figure 4), sequences that encode SUMFs which modify the C residue to FGly in the active site of FGly-SULFs were searched among the algal genomes. Table 3 shows that SUMF sequences that were highly similar to those of eukaryotic SUMFs were only retrieved from brown algae (*Ph. tricornutum, T. pseudonana*, and *E. siliculosus*) and a green microalga (plankton), *Ostreococcus tauri*. In addition to the SUMF sequences, SUMF-like sequences that are highly similar to the coding sequence of Meiotically Up-regulated Gene (MUG) 158 (also known as Egt1) from a yeast, *Sch. pombe*, were retrieved from green (*Auxenochlorella protothecoides*), brown (*T. pseudonana* and *E. siliculosus*) and red algae (*C. crispus, Po. cruetum, Cy. merolae*, and *Ga. sulphuraria*), as well as a moss (Bryophyte), *Physcomitrella patens* (Pp1s94_113V6 abbreviated as PHYPA_113V6) which represents the missing link between green algae and higher land plants (Figure 5).

**Table 3:** **The algal sequences encoding SUMFs and SUMF-like proteins**.

**Species**	**Accession Numbers**		
	**UniprotKB**	**Enesmbl Genomes**	**Identifier**
**SUMF**			
*Thalassiosira pseudonana*	B8BTF8	Thaps3|2123_fgenesh1_pg_C_chr_2000078; THAPSDRAFT_261211	THAPS_ B8BTF8
*Phaeodactylum tricornutum*	–	PHATRDRAFT_bd1393	PHATR_B7S479
*Ectocarpus siliculosus*	D8LGF4	Esi_0167_0035	ECTSI_D8LGF4
	D8LGF5	Esi_0167_0037	ECTSI_D8LGF5
	D7FPR0	Esi_0195_0042	ECTSI_D7FPR0
*Ostreococcus tauri*	–	Ostta4|12317|fgenesh1_pg.C_Chr_09.0001000147	OSTTA_12317
**SUMF-LIKE**			
*Thalassiosira pseudonana*	B8C863	Thaps3|8019_fgenesh1_pg_C_chr_9000187; THAPSDRAFT_8019	THAPS_ B8C863
*Ectocarpus siliculosus*	D7FTN7	Esi_0253_0004	ECTSI_D7FTN7
*Auxenochlorella protothecoides*	A0A087SAC0	–	AUXPR_ A0A087SAC0
*Cyanidioschyzon merolae*	M1UW85	CYME_CMR147C	CYAME_M1UW85
*Galderia sulphuraria*	M2X023	–	GALSU_M2X023
*Chondrus crispus*	R7QHQ9	–	CHOCR_R7QHQ9
*Porphyridium cruetum*	–	evm.model.contig_3699	PORCR_contig3699

The SUMF-like sequences were longer than the SUMF sequences. These two groups of sequences are only identical at the formyl-glycine generating enzyme (FGE)-sulfatase domain (pfam 03781). MUG 158 from *Sch. pombe* has an S-adenosyl-L-methionine (SAM)-dependent methyltransferase domain (pfam 10017; including DUF 2260, a domain with unknown function), an uncharacterized DinB_2 domain (pfam 12867; including an iron-binding motif, H-X(3)-H-X-E), in addition to the FGE-sulfatase domain (Pluskal et al., 2014). This protein was reported to be involved in cell division and its expression was up-regulated upon the entry of cell into meiosis (Mata et al., 2002). Highly identical to the sequences of NcEgt1 from *N. crassa* and MsEgtD from *Mycobacterium smegmatis*, MUG 158 was also reported to be involved in the first step of ergothioneine biosynthesis (Pluskal et al., 2014). Ergothioneine, an amino acid derived from thiourea that contains components associated with histidine, was reported to accumulate in oxidative-stress susceptible area in human body (Cheah and Halliwell, 2012) thus was believed to be able to scavenge oxidizing species that are not free radicals (Chaudière and Ferrari-Iliou, 1999). However, ergothioneine is only synthesized by a few filamentous fungi, actinobacteria, and cyanobacteria but not by higher plants and animals. The red alga, *Po. purpureum* SAG1380-1C, was reported to produce a small amount of ergothioneine (Saha et al., 2015). It is unknown whether the algal SUMF-like sequences share the same function as SUMF sequences, or have other functions in ergothionein biosynthesis or meiosis as in MUG 158. Alternatively, these sequences may possess both functions. At least three brown algae were found to have both SUMF and SUMF-like sequences, indicating that both types of sequences could have different functions.

**Figure 6:**
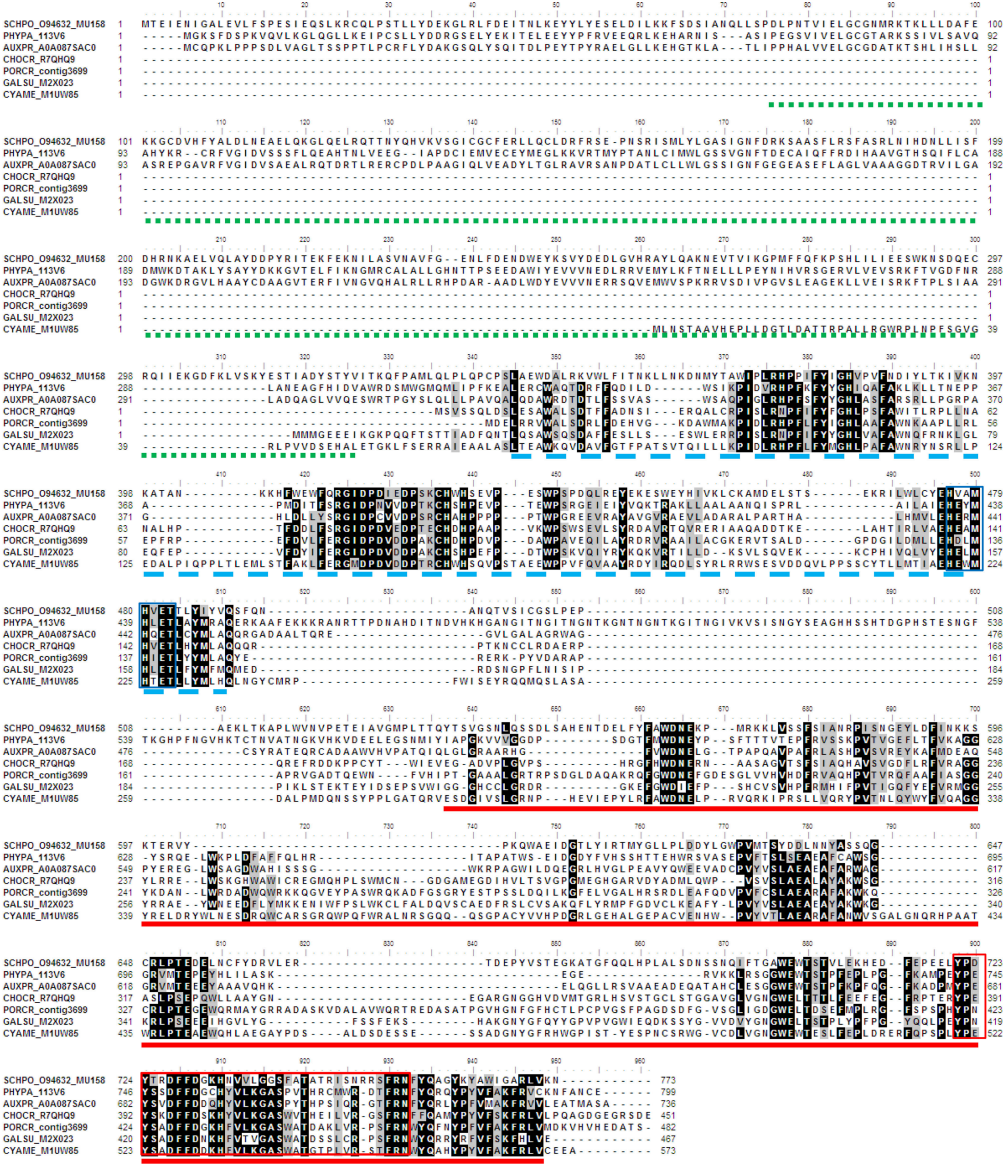
**Multiple sequence alignment of algal SUMF-like sequences**. The amino acid sequences were aligned by ClustalW. Identical and similar sequences were highlighted in black and gray, respectively. The pfam domains 10017 (Histidine-specific SAM-dependent methyltransferase), 12867 (DinB domain), and 037181 (FGE-sulfatase) are underlined with green (dotted line), blue (broken line) and red, respectively. The DinB_2 iron-binding motif is indicated by blue box while the red box shows the EgtB subfamily C-terminal sequences. The identifier of the sequence starts with the species abbreviation followed by the UNIPROT/Genbank accession number and annotation wherever possible (Table 2). PHYPA, *Physcomitrella patens;* SCHPO, *Schizosaccharomyces pombe*.

The phylogeny of SUMF and SUMF-like sequences (Figure 5) shows two main clusters consisting of SUMF sequences and SUMF-like sequences, respectively; which may share the same ancestor. The SUMF cluster consists of human SUMF1-3, bovin SUMF 1-2, mouse SUMF 1-2 together with four SUMFs from three brown algae (one from *Ph. tricornutum* and *T. pseudonana*, respectively; two from *E. siliculosus*) and one from the green microalga *O. tauri*. Each of the SUMF sequences in this cluster contains a FGE-sulfatase domain except for one of the SUMF sequences from *E. siliculosus* (ECTSI_D8LGF4) which has an incomplete domain while the SUMF sequence from *O. tauri* has an additional but incomplete glycosyltransferase domain (pfam 00534). The SUMF-like cluster consists of MUG 158 from *Sch. pombe*, SUMF-like sequences from *A. protothecoides, T. pseudonana, E. siliculosus, C. crispus, Po. cruetum, Cy. merolae, Ga. sulphuraria*, and *Phy. patens*. The domains found in the SUMF-like sequences are more variable. The red algal SUMF-like sequences were found to contain DinB_2 domain (pfam 12867) at their N-termini, in addition to the FGE-sulfatase domain (Figures 5, 6). The SUMF-like sequences from the green lineage (moss and green alga), similar to MUG158, were found to have two additional domains, i.e., pfam 10017 (S-adenosyl-L-methionine (SAM)-dependent methyltransferase domain) and pfam 12867 at the N-terminus of the FGE-sulfatase domain; while the brown algal SUMF-like sequences have pfam 12867 and pfam 10017 at the N- and C-termini of FGE-sulfatase domain, respectively (Figure 6). One of the sequences from *E. siliculosus* (ECTSI_D8LGF5) which has an incomplete FGE-sulfatase domain could not be assigned to either group of sequences.

It is intriguing that SUMF sequences were not found in the genomes of both green algae *Ch. reinhardtii* and *V. carteri* which have FGly-SULF sequences; and equally intriguing that SUMF sequence was found in *O. tauri* wherein FGly-SULF sequence was not detected. Similarly, SUMF or SUMF-like sequences were not reported in *Saccharomyces cerevisiae* and a few other yeasts which were shown to have FGly-SULFs. Could there be other sequences that are able to modify FGly-SULFs in *Ch. reinhardtii* and *V. carteri*? Alternatively, modification of Cys to FGly may not be necessary for these green algal FGly-SULFs. It is obvious that a group of SUMF-like sequences are present in green, brown, and red algae as well as moss, yeasts (at least Saccharomycetes and Ascomycetes), bacterium *Mycobacterium*, however, their functions are uncharacterized.

In general, the phylogeny of algal CHSTs, FGly-SULFs, and SUMFs or SUMF-like sequences revealed that many protein sequences were clustered according to their groups i.e., green (for CHSTs with pfam Sulfotransfer_1 domain), brown (for CHSTs, FGly-SULFs, and SUMFs or SUMF-like sequences), and red (for CHSTs with pfam Sulfotransfer_2 domain, FGly-SULFs and SUMFs or SUMF-like sequences) algae. Duplication/multiplication and functional divergence of these sequences could have happened after the divergence of these three groups of algae during evolution. Since only two green algal SUMFs or SUMF-like sequences were retrieved, the same trend was not observed. The clustering of a few CHSTs with pfam Sulfotransfer_1 domain from different groups of algae implied the existence of an ancestral sequence before the separation of these algal groups. The phylogenetic analyses of these putative genes contribute to the corpus of knowledge of an unexplored area. Algal CHSTs, FGly-SULFs, and SUMFs constitute a highly attractive target for future research to produce existing and new sulfated carbohydrate polymers through enzymatic synthesis and metabolic engineering.

### Conflict of interest statement

The author declares that the research was conducted in the absence of any commercial or financial relationships that could be construed as a potential conflict of interest.

## References

[B1] Altschul S. F. Gish W. Miller W. Myres E. W. Lipman D. J. (1990). Basic local alignment search tool. J. Mol. Biol. 215, 403–410. 10.1016/S0022-2836(05)80360-2 2231712

[B2] Aquino R. S. Landeira-Fernandez A. M. Valente A. P. Andrade L. R. Mourão P. A. S. (2005). Occurrence of sulfated galactans in marine angiosperms: evolutionary implications. Glycobiology 15, 11–20. 10.1093/glycob/cwh138 15317737

[B3] Armbrust E. V. Berges J. A. Bowler C. Green B. R. Martinez D. Putnam N. H.. (2004). The genome of the diatom *Thalassiosira pseudonana*: ecology, evolution, and metabolism. Science 306, 79–86. 10.1126/science.1101156 15459382

[B4] Baum H. Dodgson K. S. (1957). Differentiation between myrosulphatase and the arylsulphatases. Nature 179, 312–313. 10.1038/179312a0 13407709

[B5] Bhattacharya D. Price D. C. Chan C. X. Qiu H. Rose N. Ball S.. (2013). Genome of the red alga *Porphyridium purpureum*. Nat. Commun. 4, 1941. 10.1038/ncomms2931 23770768 PMC3709513

[B6] Bochenek M. Etherington G. J. Koprivova A. Mugford S. T. Bell T. G. Malin G.. (2013). Transcriptomic analysis of the sulfate deficiency response in the marine microalga *Emiliania huxleyi*. New Phytol. 199, 650–662. 10.1111/nph.12303 23692606

[B7] Boltes I. Czapinska H. Kahnert A. von Bülow R. Dierks T. Schmidt B.. (2001). 1.3 A structure of arylsulfatase from *Pseudomonas aeruginosa* establishes the catalytic mechanism of sulfate ester cleavage in the sulfatase family. Structure 9, 483–491. 10.1016/S0969-2126(01)00609-8 11435113

[B8] Bowler C. Allen A. E. Badger J. H. Grimwood J. Jabbari K. Kuo A. (2008). The *Phaeodactylum* genome reveals the dynamic nature and multi-lineage evolutionary history of diatom genomes. Nature 456, 239–244. 10.1038/nature07410 18923393

[B9] Chaudière J. Ferrari-Iliou R. (1999). Intracellular antioxidants: from chemical to biochemical mechanisms. Food Chem. Toxicol. 37, 949–962. 10.1016/S0278-6915(99)00090-3 10541450

[B10] Cheah I. K. Halliwell B. (2012). Ergothioneine; antioxidant potential, physiological function and role in disease. Biochim. Biophys. Acta 1822, 784–793. 10.1016/j.bbadis.2011.09.017 22001064

[B11] Chenna R. Sugawara H. Koike T. Lopez R. Gibson T. J. Higgins D. G.. (2003). Multiple sequence alignment with the Clustal series of programs. Nucleic Acids Res. 31, 3497–3500. 10.1093/nar/gkg500 12824352 PMC168907

[B12] Cock J. M. Sterck L. Rouzé P. Scornet D. Allen A. E. Amoutzias G.. (2010). The *Ectocarpus* genome and the independent evolution of multicellularity in brown algae. Nature 465, 617–621. 10.1038/nature09016 20520714

[B13] Collén J. Porcel B. Carré W. Ball S. G. Chaparro C. Tonon T.. (2013). Genome structure and metabolic features in the red seaweed *Chondrus crispus* shed light on the evolution of the Archaeplastida. Proc. Natl. Acad. Sci. U.S.A. 110, 5247–5252. 10.1073/pnas.1221259110 23503846 PMC3612618

[B14] DeBoer J. A. (1981). Nutrients, in The Biology of Seaweeds, eds Lobban C. S. Wynne M. J. (Oxford: Blackwell Scientific), 359–391.

[B15] Dierks T. Lecca M. R. Schmidt B. von Figura K. (1998a). Conversion of cysteine to formylglycine in eukaryotic sulfatases occurs by a common mechanism in the endoplasmic reticulum. FEBS Lett. 423, 61–65. 10.1016/S0014-5793(98)00065-9 9506842

[B16] Dierks T. Miech C. Hummerjohann J. Schmidt B. Kertesz M. A. von Figura K. (1998b). Posttranslational formation of formylglycine in prokaryotic sulfatases by modification of either cysteine or serine. J. Biol. Chem. 273, 25560–25564. 10.1074/jbc.273.40.25560 9748219

[B17] Dierks T. Schmidt B. von Figura K. (1997). Conversion of cysteine to formylglycine: a protein modification in the endoplasmic reticulum. Proc. Natl Acad. Sci. U.S.A. 94, 11963–11968. 10.1073/pnas.94.22.11963 9342345 PMC23670

[B18] Ehrhardt D. W. Atkinson E. M. Faull K. F. Freedberg D. I. Sutherlin D. P. Armstrong R.. (1995). *In vitro* sulfotransferase activity of NodH, a nodulation protein of *Rhizobium meliloti* required for host-specific nodulation. J. Bacteriol. 177, 6237–6245. 7592390 10.1128/jb.177.21.6237-6245.1995 PMC177465

[B19] Felsenstein J. (1985). Phylogenies and the comparative method. Am. Nat. 125, 1–15. 10.1086/284325

[B20] Friedlander M. (2001). Inorganic nutrition in pond cultivated *Gracilaria conferta* (Rhodophyta): nitrogen, phosphate and sulfate. J. Appl. Phycol. 13, 278–296. 10.1023/A:1011139329415

[B21] Fukuda M. Hiraoka N. Akama T. O. Fukuda M. N. (2001). Carbohydrate-modifying sulfotransferases: structure, function, and pathophysiology. J. Biol. Chem. 276, 47747–47750. 10.1074/jbc.R100049200 11585845

[B22] Gao Y. Schofield O. M. E. Leustek T. (2000). Characterization of sulfate assimilation in marine algae focusing on the enzyme 5′-adenylylsulfate reductase. Plant Physiol. 123, 1087–1096. 10.1104/pp.123.3.1087 10889258 PMC59072

[B23] Genicot-Joncour S. Poinas A. Richard O. Potin P. Rudolph B. Kloareg B. Helbert W. (2009). The cyclization of the 3,6-anhydro-galactose ring of ?-carrageenan is catalyzed by two ?-galactose-2,6-sulfurylases in the red alga *Chondrus crispus*. Plant Physiol. 151, 1609–1616. 10.1104/pp.109.144329 19734263 PMC2773109

[B24] Gijsbers R. Ceulemans H. Stalmans W. Bollen M. (2001). Structural and catalytic similarities between nucleotide pyrophosphatases/phosphodiesterases and alkaline phosphatases. J. Biol. Chem. 276, 1361–1368. 10.1074/jbc.M007552200 11027689

[B25] Hagelueken G. Adams T. M. Wiehlmann L. Widow U. Kolmar H. Tümmler B.. (2006). The crystal structure of SdsA1, an alkylsulfatase from *Pseudomonas aeruginosa*, defines a third class of sulfatases. Proc. Natl. Acad. Sci. U.S.A. 103, 7631–7636. 10.1073/pnas.0510501103 16684886 PMC1472496

[B26] Hanin M. Jabbouri S. Quesada-Vincens D. Freiberg C. Perret X. Promé J. C.. (1997). Sulphation of *Rhizobium* sp. NGR234 Nod factors is dependent on *noe*E, a new host-specificity gene. Mol. Microbiol. 24, 1119–1129. 10.1046/j.1365-2958.1997.3981777.x 9218762

[B27] Hanson S. R. Best M. D. Wong C. H. (2004). Sulfatases: structure, mechanism, biological activity, inhibition, and synthetic utility. Angew. Chem. Int. Ed Engl. 43, 5736–5763. 10.1002/anie.200300632 15493058

[B28] Hernandez-Sebastia C. Varin L. Marsolais F. (2008). Sulfotransferases from plants, algae and phototrophic bacteria, in Sulfur Metabolism in Phototrophic Organisms, eds Hell R. Dahl C. Leustek T. (Dordrecht: Springer), 111–130.

[B29] Hopwood J. J. Ballabio A. (2001). Multiple sulfatase deficiency and the nature of the sulfatase family, in The Metabolic and Molecular Bases of Inherited Disease, eds Scriver C. R. Beaudet A. L. Sly W. S. Valle D. Childs B. Kinzler K. W. (New York, NY: McGraw-Hill), 3725–3732.

[B30] Kakinuma M. Kaneko I. Coury D. A. Suzuki T. Amano H. (2006). Isolation and identification of gametogenesis-related genes in *Porphyra yezoensis* (Rhodophyta) using subtracted cDNA libraries. J. Appl. Phycol. 18, 489–496. 10.1007/s10811-006-9052-8

[B31] Landgrebe J. Dierks T. Schmidt B. von Figura K. (2003). The human SUMF1 gene, required for posttranslational sulfatase modification, defines a new gene family which is conserved from pro- to eukaryotes. Gene 316, 47–56. 10.1016/S0378-1119(03)00746-7 14563551

[B32] Lee H. Lee H. K. An G. Lee Y. K. (2007). Analysis of expressed sequence tags from the red alga *Griffithsia okiensis*. J. Microbiol. 45, 541–546. 18176538

[B33] Mata J. Lyne R. Burns G. Bähler J. (2002). The transcriptional program of meiosis and sporulation in fission yeast. Nat. Genet. 32, 143–147. 10.1038/ng951 12161753

[B34] Matsuzaki M. Misumi O. Shin-I T. Maruyama S. Takahara M. Miyagishima S. Y.. (2004). Genome sequence of the ultrasmall unicellular red alga *Cyanidioschyzon merolae* 10D. Nature 428, 653–657. 10.1038/nature02398 15071595

[B35] McHugh D. J. (2003). A guide to the seaweed industry, in FAO Fisheries Technical Paper (Rome), 441.

[B36] Merchant S. S. Prochnik S. E. Vallon O. Harris E. H. Karpowicz S. J. Witman G. B.. (2007). The *Chlamydomonas* genome reveals the evolution of key animal and plant functions. Science 318, 245–250. 10.1126/science.1143609 17932292 PMC2875087

[B37] Meroni G. Franco B. Archidiacono N. Messali S. Andolfi G. Rocchi M.. (1996). Characterization of a cluster of sulfatase genes on Xp22.3 suggests gene duplications in an ancestral pseudoautosomal region. Hum. Mol. Genet. 5, 423–431. 10.1093/hmg/5.4.423 8845834

[B38] Müller I. Kahnert A. Pape T. Sheldrick G. M. Meyer-Klaucke W. Dierks T.. (2004). Crystal structure of the alkylsulfatase AtsK: insights into the catalytic mechanism of the Fe(II) alpha-ketoglutarate-dependent dioxygenase superfamily. Biochemistry 43, 3075–3088. 10.1021/bi035752v 15023059

[B39] Nikaido I. Asamizu E. Nakajima M. Nakamura Y. Saga N. Tabata S. (2000). Generation of 10,154 expressed sequence tags from a leafy gametophyte of a marine red alga, *Porphyra yezoensis*. DNA Res. 7, 223–227. 10.1093/dnares/7.3.223 10907854

[B40] Opoku G. Qiu X. Doctor V. (2006). Effect of oversulfation on the chemical and biological properties of kappa carrageenan. Carbohyd. Polym. 65, 134–138. 10.1016/j.carbpol.2005.12.033

[B41] Parenti G. Meroni G. Ballabio A. (1997). The sulfatase gene family. Curr. Opin. Genet. Dev. 7, 386–391. 10.1016/S0959-437X(97)80153-0 9229115

[B42] Pluskal T. Ueno M. Yanagida M. (2014). Genetic and metabolomic dissection of the ergothioneine and selenoneine biosynthetic pathway in the fission yeast, *S. pombe*, and construction of an overproduction system. PLoS ONE 9:e97774. 10.1371/journal.pone.0097774 24828577 PMC4020840

[B43] Pomin V. H. Mourão P. A. (2008). Structure, biology, evolution and medical importance of sulfated fucans and galactans. Glycobiology 18, 1016–1027. 10.1093/glycob/cwn085 18796647

[B44] Poux N. (1966). Ultrastructural localization of aryl sulfatase activity in plant meristemic cells. J. Histochem. Cytochem. 14, 932–933. 10.1177/14.12.932 17121393

[B45] Qin X. Ma C. Lou Z. Wang A. Wang H. (2013). Purification and characterization of ?-Gal-6-sulfurylasesfrom *Eucheuma stratrium*. Carbohyd. Polym. 96, 9–14. 10.1016/j.carbpol.2013.03.061 23688448

[B46] Rees D. A. (1961a). Enzymic desulphation of porphyran. Biochem. J. 80:449. 10.1042/bj0800449 13740282 PMC1243251

[B47] Rees D. A. (1961b). Enzymic synthesis of 3:6-andydro-L-galactose within porphyran from L-galactose 6-suphate units. Biochem. J. 81:347. 10.1042/bj0810347 16748934 PMC1243346

[B48] Saha S. K. McHugh E. Murray P. Walsh D. J. (2015). Chapter 12 Microalagae as a source of nutraceuticals, in Phycotoxins: Chemistry and Biochemistry, 2nd Edn., eds Botana L. M. Alfonso A. (Chichester: John Wiley & Sons, Ltd), 271.

[B49] Saitou N. Nei M. (1987). The neighbor-joining method: a new method for reconstructing phylogenetic trees. Mol. Biol. Evol. 4, 406–425. 3447015 10.1093/oxfordjournals.molbev.a040454

[B50] Sardiello M. Annunziata I. Roma G. Ballabio A. (2005). Sulfatases and sulfatase modifying factors: an exclusive and promiscuous relationship. Hum. Mol. Genet. 14, 3203–3217 10.1093/hmg/ddi351 16174644

[B51] Schirmer A. Kolter R. (1998). Computational analysis of bacterial sulfatases and their modifying enzymes. Chem. Biol. 5, R181–R186. 10.1016/s1074-5521(98)90154-5 9710560

[B52] Schmidt B. Selmer T. Ingendoh A. von Figura K. (1995). A novel amino acid modification in sulfatases that is defective in multiple sulfatase deficiency. Cell 82, 271–278. 10.1016/0092-8674(95)90314-3 7628016

[B53] Shukla M. K. Kumar M. Prasad K. Reddy C. R. K. Jha B. (2011). Partial characterization of sulfohydrolase from *Gracilaria dura* and evaluation of its potential application in improvement of the agar quality. Carbohyd. Polym. 85, 157–163. 10.1016/j.carbpol.2011.02.009

[B54] Tamura K. Dudley J. Nei M. Kumar S. (2007). MEGA4: MOLECULAR Evolutionary Genetics Analysis (MEGA) software version 4.0. Mol. Biol. Evol. 24, 1596–1599. 10.1093/molbev/msm092 17488738

[B55] Teo S.-S. Ho C.-L. Teoh S. Lee W.-W. Tee J.-M. Raha A. R. (2007). Analyses of expressed sequence tags from an agarophyte, *Gracilaria changii* (Gracilariales, Rhodophyta). Eur. J. Phycol. 42, 41–46. 10.1080/09670260601012461

[B56] Tuvikene R. Truus K. Kollist A. Volobujeva O. Mellikov E. Pehk T. (2008). Gel-forming structures and stages of red algal galactans of different sulfation levels. J. Appl. Phycol. 20, 527–535. 10.1007/s10811-007-9229-9

[B57] Wang A. Islam M. N. Qin X. Wang H. Peng Y. Ma C. (2014). Purification, identification and characterization of D-galactose-6-sulfurylase from marine algae (*Betaphycus gelatinus*). Carbohydr. Res. 388, 94–99. 10.1016/j.carres.2013.12.010 24632215

[B58] Wong K. F. Craigie J. S. (1978). Sulfohydrolase activity and carrageenan biosynthesis in *Chondrus crispus* (Rhodophyceae). Plant Physiol. 61, 663–666. 10.1104/pp.61.4.663 16660358 PMC1091939

[B59] Yildiz F. H. Davies J. P. Grossman A. R. (1994). Characterization of sulfate transport in *Chlamydomonas reinhardtii* during sulfur-limited and sulfur-sufficient growth. Plant Physiol. 104, 981–987. 12232142 10.1104/pp.104.3.981 PMC160696

